# The Hospital. Nursing Section

**Published:** 1902-06-07

**Authors:** 


### The Hospital.

### Nursing Section.

Contributions for this Section of "The Hospital" should be addressed to the Editor, "The Hospital" Nursing Section, 28 & 29 Southampton Street, Strand, London, W.C.

No. 819.—Vol. XXXII. *SATURDAY, JUNE* 7, 1902.

### Notes on News from the Nursing World.

### 1856 AND 1902.

The glad tidings flashed to earth's remotest limits on Sunday evening, that terms of surrender had been signed at Pretoria, will of course be followed by the return of most of the members of the Queen Alexandra's Military Nursing Service. It is inevitable that in this connection our thoughts should go back to the debate in Parliament at the close of the Crimean War, memorable to nurses through all time because it was then that Lord Ellesmere, moving the address upon the treaty of peace paid his splendid tribute to Miss Florence Nightingale. "The angel of mercy," he said. "still lingers to the last on the scene of her labours, but her mission is all but accomplished. Those long arcades of Scutari, in which dying men sat up to catch the sound of her footstep or the flutter of her dress, and fell back on their pillows content to have seen her shadow as it passed, are now comparatively deserted." There were "angels of mercy" too in the hospitals on the South African veld, and they also will be quite content to remain where they are so long as there is work to be done. But in the meanwhile their ministrations are entitled to the fullest recognition.

### JUDICIAL CENSURE OF THE LAMBETH GUARDIANS.

A very strong opinion was expressed from the bench respecting the conduct of the Lambeth Guardians concerning Miss Rosalie Mansell, who has obtained £600 damages for an outrageous libel. After refusing to stay execution, asked for on the pretext that the award was excessive, and observing that he would not have been surprised if higher damages had been given, Mr. Justice Grantham proceeded to hear the action which Miss Mansell brought against Mr. and Mrs. Ayles, the master and matron of the Renfrew Road Workhouse. It was urged on behalf of the defendants that they never intended to make a statement to the Board of Guardians reflecting on Miss Mansell's character. All they did was to report a certain statement made by an inmate. They did not say there was any truth whatever in what the inmate said, and under the circumstances their counsel asked that a juror should be withdrawn. Mr. Lush, K.C., for the plaintiff, said that her only object was to clear herself, and that that object was now attained. In these circumstances, the judge assented to the application, remarking, however, that "the conduct of the Guardians was extraordinary, for they failed to act in a judicial capacity as a public body should do." This is a severe censure from the bench, but we are afraid that the Lambeth Guardians are far from being singular in failing to act in a judicial capacity. The average board has not yet risen to such a high conception of duty.

### VISITING NURSES FOR PAYING PATIENTS.

An interesting movement has been set on foot in Marylebone with the Duchess of Fife as president. The object is "to provide a visiting nurse to attend paying patients in the district by the hour." This visiting nurse is to work under the doctors of the district, and her charges are to be 2s. 6d. a visit, 12s. 6d. a week, or 5s. for an emergency visit. The idea of visiting nurses for paying patients is, as we have said before, excellent, if it can be carried out in a satisfactory manner. But to provide one visiting nurse only for such a huge borough as Marylebone is not sufficient, even by way of experiment. As to charges, if a nurse can visit a number of patients in the blocks of flats which pervade Marylebone, half-a-crown is a reasonable fee for her services. It is not enough if she has to walk half a mile, from one patient to another. Unless the managers of the new association can decide to employ more than one nurse, they would do well to limit her visits to a portion of the district; and if she obtains adequate support, they can easily extend the operations to the remainder by means of a sufficient staff.

### HOURLY NURSING IN AMERICA.

By the way, the branch of work known as hourly nursing is developing in America. A pioneer of the movement, who started the work of hourly nursing in June 1896, achieved success in the following manner, and her experience is now published for the benefit of others who desire to labour in the same field. She called on all the doctors she knew and explained her scheme. To bring it before those with whom she was not acquainted, she had cards printed under the title of visiting nurse, with schedule of charges as follows:—One or two hours night and morning, a dollar a day; prepare for minor operation and assist, two dollars; remain with patient all night, three dollars; obstetrical cases, to be present during labour, six hours or less, two dollars; and 25 cents additional for every two hours longer up to five dollars. That these charges commended themselves to the American public may be judged by the fact that the nurse's experiment has been attended by marked financial success.

### THE JOHANNESBURG CO-OPERATION.

It may interest some of our readers to learn that the fees for fully-trained hospital nurses belonging to the newly-started nurses' co-operation at Johannesburg are from five to seven guineas per week; and the fees per day, or per night, a guinea. The nurses can be accommodated at the Co-operation Residential Club with a furnished room at 3s. 6d. per night, or from £2 3s. to £6 6s. per month. For meals they are charged 6s. per day, or breakfast 1s. 6d., dinner 2s. 6d., luncheon 2s., no charge being made for early or afternoon tea. The club subscription is a guinea per annum. The percentage on the fees is 1s. 6d. in the £, or 7s. 6d. if £5 5s. a week are earned. We are informed that there is an opening for good nurses in Johannesburg, but we advise those who think of going out to any part of South Africa to make independent inquiries before they decide to start.

### SPECIAL TRAINING AT THE ANTIPODES.

The Committee of the Women's Hospital at Melbourne have under consideration a proposal from the president of the Victorian Trained Nurses' Association—who are arranging for the attainment of an accepted standard of education for nurses throughout Victoria—that that institution should be recognised as a special training school in midwifery and gynæcology. The association now consists of 350 nurses, and is in reciprocal relationship with the Sydney Association. The two bodies are recognised throughout the Commonwealth, and the training of all nurses belonging to them comprises a three years' curriculum in hospitals at which a systematic course of lectures is given. At present the Women's Hospital trains its own nurses generally, but if the proposal of the Association is accepted, the training will be exclusively confined to midwifery and gynæcology.

### THE WHITBY ISOLATION HOSPITAL.

We understand that within the last fortnight a wooden building has been erected at Whitby Isolation Hospital to contain eight or twelve beds, but "that no alterations have been effected in the sanitary arrangements or in the nurses' sleeping quarters." The authorities, we are not surprised to hear, are much annoyed that it is now known that scarlet fever and diphtheria prevail in the town. A nurse at the Isolation Hospital is now reported to have "a bad sore throat," a very natural consequence if she sleeps in the diphtheria ward. As it appears that the authorities imagine our correspondent is one of the nurses in their employ, we think it only fair to state that the letter did not emanate from any member of the staff.

### A NEW HOME AT WAKEFIELD.

The nurses' home in connection with the Clayton Hospital at Wakefield, which has just been opened, is a very handsome building. It comprises a commodious dining-hall, reception-room, 20 bedrooms, bath-rooms, and box-rooms. The exterior is of Elland Edge wall-stone with Huddersfield stone dressings, and is in the Flemish Gothic style of architecture, harmonising with the older portion of the building. The nurses of the hospital owe to a bequest of the late Mr. Joseph Shaw the exceedingly good accommodation which they now enjoy.

### THE NURSES OF THE BRITISH LYING-IN HOSPITAL.

A special appeal has been issued by the Duke of Portland for funds to enable the committee of the British Lying-in Hospital to build a nurses' home. At present some of the nurses are accommodated in a house in Little Russell Street, and "it is not right," said one of the trustees at a special meeting in January this year, "that ladies should have to turn out late at night in all sorts of weather." At that meeting plans for pulling down a house belonging to the hospital and erecting a nurses' home on the site were approved, and the committee were empowered to spend £7,000, £600 of which was for furnishing. The house, No. 28 Betterton Street, has now been pulled down, 14 feet have been dug out for cellarage, and the walls are being underpinned. It is hoped that the home will be completed early in 1903; it will be connected with the main building, and in many ways there will be a saving of labour to the nursing staff. The appeal states that accommodation will be provided in the home for twenty nurses and midwives, and that this will secure the opening of two fresh wards. One of these is at present occupied by four of the nurses.

### THE WARWICK NURSING ASSOCIATION.

Like a wise man, Lord Warwick, before he took the chair at the annual meeting of the Warwick Nursing Association, endeavoured to obtain from his wife, who unfortunately was too ill to be present, some information as to its work, and he appeared from his speech to have been excellently coached. To a certain extent Lord Warwick is right in contending that the organisation should be self supporting. That is to say, those who profit by the services of the nurses should subscribe towards it however little they can afford. In this case the friendly societies contributed £10. But, as to the general principle, Queen's nurses are the representatives of one of the noblest of charities, and Lord Warwick can scarcely mean that the poor among whom they work should find the money for their salaries. The Warwick Association is financially in a satisfactory position, the receipts for last year exceeding the expenses by £33. But among the former were £60, half the proceeds of a charity ball, and a legacy of £20, which cannot be relied upon to come in every year.

### A MIDWIFE OF 73.

An inquest was held on Friday last week by the county coroner at Stonehouse on twin children, when the following evidence was given:—Jane Esther Lamble, aged 73, midwife, and wife of John Lamble, a mason, stated that on the previous Thursday she attended Mrs. Elson, who was confined of twins. The registrar refused to grant her a certificate to bury the children as stillborn. She considered that she was a competent midwife, having had 30 years' experience. The registrar said that Mrs. Lamble was aged, and that about three months ago, when she twice applied for certificates for burial, he told her he should not grant them in future, but refer the matter to the coroner. Dr. Corbett stated that the children were stillborn, but added that the mother had not been skilfully attended to, and that medical assistance should have been obtained. The jury in returning a verdict "stillborn," expressed the opinion that the age of Mrs. Lamble militated against her undertaking such cases without medical assistance.

### REGISTRATION AND CHARACTER.

It was unfortunate that Miss Stevenson, of the Royal Infirmary, Edinburgh, the President of the Society for the State Registration of Trained Nurses, was not well enough to fulfil her engagement to take the chair at the first meeting on Friday. A letter from her was read by Miss Stewart, the matron of St. Bartholomew's Hospital, who presided in her stead. We note that Miss Stewart herself, while arguing in favour of a State register, admitted that there are "many difficulties" in the way. These, she thinks, may be overcome. The most formidable of all obstacles was not mentioned by any of the speakers. But it was very apparent at the meeting. The small attendance and the absence of the vast majority of matrons of any distinction, show how utterly the movement has failed to obtain support in the nursing world. Everyone concerned to promote the interest of nurses is as anxious as Miss Mollett "to squeeze out the inefficients." But there is a better way of doing this than by registration, which if it did, to some extent, shut out the incapables, would give a yet worse type, the nurses destitute of character, a power that would not fail to be used to the detriment of the profession.

### GOLD MEDALS FOR MONTREAL NURSES.

At the presentation of certificates, or, as they are called in Canada, diplomas, to the probationers of the Montreal General Hospital on April 28th they were given, in addition, handsome gold medals, the name of each recipient being engraved upon the medal and embossed upon the case containing it. To Mr. Crathens, the president of the hospital, the eight nurses who passed their final examination successfully are indebted for the possession of a token, which is not usually bestowed in such cases.

### THE "DON'TS" OF PRIVATE NURSING.

Following the series called "The Nurse's Never," which appeared in our columns last October from the pen of a medical lecturer on nursing, Miss Emily F. Stewart Brown, lady president for 1902 of the Hospital for Women at Liverpool, has taken "Don'ts" as the subject of a short address to nurses. The Queen has accepted a copy of the address, which contains several exhortations more or less needed, such as "Don't have crackly skirts or squeaky shoes." "Don't forget to ask if your patient has false teeth, and to have them removed before anæsthetics are given." "Don't give details to the doctor in the patient's hearing." "Don't contradict or argue with a patient." "Don't cultivate a love for the succulent onion." "Don't beat an egg in your patient's room." Certainly, these and the other "Don'ts" bear very distinctly on the little niceties of private nursing, though a nurse who has been carefully trained, and has profited by her training, ought not to need most of the warnings given.

### THE NURSING MOVEMENT IN WEST BROMWICH.

It will be remembered that some time ago we announced that Mr. Alderman Akrill, of West Bromwich, had offered to erect a nurses' home in West Bromwich at a cost of £2,000. The building has now been completed, and was opened by Lord Dartmouth last week. Unfortunately, the generous donor was absent from the ceremony owing to illness, but Mr. J. A. Kenrick, one of the speakers, having referred to the inception of the nursing work in the town some three years ago, and its rapid growth, said that everyone interested in the movement was indebted to Mr. Akrill for providing the nurses with a permanent home. He mentioned that nearly the whole of the £1,000 necessary for the movement had been raised, and though £500 of this had been spent in furnishing the home, he did not anticipate any difficulty in again making up the amount. Lord Dartmouth, in acknowledging a vote of thanks, emphasised the importance of the work of the nurses. "No movement that had been inaugurated during the last century," he said, "had made such rapid advances as that associated with nursing institutions, and in thickly-populated districts the system of trained nurses, he was pleased to see, was being very favourably received." The nurses are highly gratified with their new home.

### PROPOSED INTRODUCTION OF MALE NURSES AT A WORKHOUSE INFIRMARY.

It has somewhat reluctantly been determined by the St. Germans Board of Guardians to add two efficient nurses to the infirmary staff. Dr. Vintner, the medical officer, who in his report to the House Works Committee recommended this step, was taken to task by one of the guardians for his recommendation. But, as Dr. Vintner rejoined, he is "supposed" to make recommendations to the board, and though they are not under an obligation to always act upon them, it is exceedingly ungracious to complain of the medical officer when he suggests changes which he believes are needed. A proposal that male nurses at a salary of £20 and rations should be employed met with scanty support, and the only plea for the innovation was the saving of about £10. No attempt was made to advocate it on its merits.

### INADEQUATE ACCOMMODATION AT MORPETH.

At the annual meeting of the Morpeth Cottage Hospital and District Nursing Association, it was stated that the number of visits paid by the district nurse last year was 5,422, as against 3,763 in the previous year. This increase was partly due to a recent epidemic of enteric fever. Unfortunately, the cottage hospital is found to be increasingly unsuitable for the purpose, and the accommodation for nurses, as well as for patients, is inadequate. A new building is a felt want in the town, and we note that the fact of there being for the first time an adverse balance on the outgoings last year, does not seem likely to hinder the Association from making an attempt to obtain it.

### OUR CORONATION HOSPITAL SUNDAY NUMBER.

We remind our readers that a double number of The Hospital will be issued next Saturday, June 14th, the price of which will be 4d., instead of 2d., as usual. This number will contain two excellent photogravure plates, being portraits of the Prince and Princess of Wales, produced from new portraits of their Royal Highnesses, with autographic signatures. The plates are suitably mounted for framing. Subscribers desiring to obtain these plates must send two penny stamps before June 11th, to the Manager of The Hospital, 28 & 29 Southampton Street, Strand, W.C., as they will not otherwise be issued with subscription copies.

### SHORT ITEMS.

The final meeting of the workers of the Kensington Branch of the Queen's Nurses' Endowment Fund took place last week at the residence of the Mayor of Kensington, when it was announced that the net total collected amounted to £1,632 19s. The sum of £677 4s. has been collected in the county of Westmorland towards the same fund.—The proceeds of the sale of tickets for witnessing the Coronation Procession at the stand erected by the authorities of Charing Cross Hospital will go towards defraying the cost of the new Nurses' Home, now in course of erection

### Lectures on Gynæcology for Nurses.

By Robert Jardine, M.D., M.R.C.S., F.F.P. and S.G., F.R.S.E., Senior Physician to the Glasgow Maternity Hospital Examiner in Midwifery to the University of Glasgow.

### LECTURE XII.—PELVIC PERITONITIS.

(*Continued from Page* 109.)

*Diagnosis.*—In the description we have sufficiently indicated the distinguishing characteristics. The only thing it is likely to be confounded with is cellulitis, and we shall now indicate the chief points of difference. In peritonitis the pain is more severe; the patient lies with both legs drawn up; the effusion is much flatter and situated round the uterus, or if it is serous it will bulge behind the uterus. Vomiting is much more severe in peritonitis than in cellulitis. There are other points, but it would only confuse you to enumerate them. As a matter of fact, it is sometimes exceedingly difficult to come to an accurate diagnosis between the two, and in some cases they undoubtedly co-exist. This is of less consequence since the treatment of both conditions is very much the same.

*Prognosis.*—This is more serious than for cellulitis both as regards fatality and bad after-effects. The adhesions which are left behind may permanently displace the uterus or so bind down the tubes and ovaries as to cause sterility. In some cases the adhesions may so interfere with the normal expansion of the uterus when conception occurs that an abortion is caused. In other cases pregnancy may not be interrupted but the patient may suffer great pain, evidently from the tearing up of the adhesions as the uterus enlarges. If adhesions have formed round the bowel intestinal obstruction may result.

*Treatment.*—Preventative treatment consists in asepsis in midwifery and gynæcological work and in the careful treatment of all cases of gonorrhœa. Unfortunately, most women who suffer from gonorrhœa do not seek treatment until the disease has extended into the uterus and tubes—in fact an attack of peritonitis is often the cause of their seeking assistance.

The treatment of acute pelvic peritonitis is very much the same as of cellulitis. The bowels should be well cleared out to begin with, and may then be left quiescent for several days. For the pain opiates will have to be used freely. Hot stupes or bran poultices are also useful. If the temperature is very high, quinine may be used. Small repeated doses of aconite are sometimes useful. Stimulation with whisky, brandy, or champagne will generally be necessary. When vomiting is severe cracked ice and brandy or champagne may be given frequently. The food must be fluid, such as milk, beef extracts, etc. For thirst, sips of very hot water often give relief. If the flatulence is troublesome, enemata will be necessary. The passage of a rectal tube will sometimes give relief.

After the acute stage is over, vaginal douching will help absorption, and the bowels must then be kept open. If the case is a prolonged one, you must carefully guard against bed-sores.

Where pus forms surgical treatment has to be adopted. The abscess may be opened through the vagina or in some cases through the abdomen. In cases of chronic or repeated attacks of peritonitis the cause will probably lie in the tubes, and to cure the disease the tubes will have to be removed.

### ENDOMETRITIS.

By this term is meant an inflammation of the endometrium or mucous lining of the uterus. The endometrium of the cervical canal as well as that of the cavity of the uterus proper is affected. Endocervicitis or inflammation of the cervical mucous membrane is often described separately, but as the two co-exist, and are practically the same, we shall consider them both under the term endometritis.

The endometrium lies so closely applied to the muscular coat of the uterus that when it is inflamed the muscular coat will also be more or less affected, *i.e.*, that with endometritis there is always more or less metritis. Various forms of endometritis are described, but we shall not attempt to differentiate between them. Generally speaking the mucous membrane is swollen and soft and covered with blood-stained mucus or pus. Small hæmorrhages will be found here and there. The glands are very active, so there is a profuse leucorrhœal discharge. Various micro-organisms may be detected in the mucous membrane and even in the muscular coat in septic cases as after abortions.

*Causes.*—In many cases direct microbic infection can be demonstrated, as in cases arising from gonorrhœa, after abortions or full-time labours, or from the passage of an unclean instrument. A chill, want of care during menstruation, and displacements of the uterus all predispose to an attack, but how far these conditions may be a direct cause it is difficult to say. Micro-organisms will probably be found to be the true cause in all cases. It arises in connection with all acute infectious diseases, such as enteric, scarlet fever, small-pox, measles, cholera, etc., and in some cases of poisoning, especially with phosphorus. It is also associated with new growths of the uterus or cervix, such as cancers or fibroids. Lacerations of the cervix by exposing a portion of the cervical mucous membrane predispose to it as infection may easily get in. It may be acute or chronic. The chronic form may follow the acute, but it may arise of itself in patients suffering from constitutional diseases, such as scrofula or chlorosis, or from a displacement.

*Symptoms.*—In an acute attack there is more or less fever, pain in the back and lower part of the abdomen with a feeling of weight in the pelvis. There is a profuse vaginal discharge, clear at first, but mixed with pus in a few days. Menstruation may be suppressed, and it is rarely increased. In the chronic form there is a very profuse leucorrhœal discharge, which is often stained with blood. Menstruation is increased and may recur frequently. There is generally more or less dysmenorrhœa, especially if there is any displacement of the uterus. The patient will complain of weakness and pain in the back and loins. Walking or standing will easily tire her. She will generally be sterile, but if she should become pregnant she will probably abort. The patient's digestive and nervous systems will become deranged. Nervous depression may amount to melancholia.

*Diagnosis.*—In the acute form there will be tenderness on pressure over the uterus, due to involvement of the muscular and peritoneal coats in the inflammatory attack. On making a vaginal examination the cervix will be felt to be swollen and the external os gaping. Round the os it will feel soft and velvety, the swollen mucous membrane of the cervix being everted. On passing a vaginal speculum the cervix will be seen to be swollen, the os gaping, and the mucous membrane round it reddened. A profuse discharge will be seen coming from the os. The discharge from the uterus proper will be watery, while that from the cervix will be thicker and very like white of egg. It is ropey and tenacious. In the septic forms, as after abortion, the discharge will be very foul and not clear. If a sound is passed it will cause pain and probably hæmorrhage.

In the chronic form a bimanual examination will reveal that the uterus is enlarged and softer than normal. The

LECTURES ON GYNÆCOLOGY FOR NURSES.—*Continued.*

sound will pass beyond the usual depth and will cause some bleeding and often considerable pain when the end of it touches the fundus. One may be able to make out with the sound that there are irregularities of the surface.

*Prognosis.*—Endometritis is not of itself a fatal disease, but it may have a very serious effect upon the patient's constitution. The excessive and repeated losses of blood may weaken the patient very much. If she becomes pregnant before the condition is completely cured an abortion is almost sure to occur.

*Treatment.*—In the acute form hot fomentations should be applied, the bowels thoroughly cleared out, and opium be given if there is much pain. Instead of hot fomentations ice bags are used by some. Vaginal douches or applications to the interior of the uterus are not to be used until the acute stage is passed. The vast majority of cases have passed the acute stage when first seen. Various methods of treatment are then adopted, such as applications of various germicidal drugs to the endometrium, packing of the uterine cavity, curetting, vaporisation of the interior of the uterus with steam, etc. Applications may be made to the interior of the uterus by swabbing it out with a dressed Playfair's probe dipped in the drug. Pure carbolic acid, a mixture of carbolic acid and iodine (iodised phenol), liniment of iodine, liquid perchloride of iron, formalin, etc., may be used in this way. Sometimes the application is made by passing the drug in in the form of a pencil or bougie, and allowing it to melt *in situ*. It is sometimes injected by means of a special syringe. The objection to the injection is that the fluid, such as Tr. of iodine, may be forced through the tubes. The injection is also liable to cause most agonising uterine colic. This may arise after an application with the probe, but not so frequently.

Packing of the uterus with a slender ribbon of iodoform gauze or of gauze saturated with carbolic acid, and leaving it *in situ* for 48 hours is strongly advocated by some. Curetting or removal of the endometrium has of recent years come very much into use. In suitable cases it is undoubtedly the best form of treatment. It is specially efficient in cases where there is much hæmorrhage.

Vaporisation or steaming the endometrium has been introduced within recent years. A special apparatus is necessary. The endometrium is destroyed by the steam. A brownish discharge, not unlike beef tea, escapes while the steam is being applied. If it is applied too long, the entire uterine cavity may afterwards close up.

Whatever method of treatment is adopted, strictest aseptic precautions must be taken. Certain drugs such as chlorate of potash, arsenic, etc., are said to have an effect on the endometrium when taken internally, but it is doubtful if they do much good. The patient's bowels should be kept free, and she should have a course of tonics. Easton's syrup is often useful. The waters of certain mineral springs seem to do good.

The patient must be warned that the treatment will extend over a considerable period, especially if applications are made to the endometrium in the usual way. She should be very careful of cold and damp at her menstrual periods, and should refrain from sexual intercourse until the cure is complete. Daily vaginal douching should be carried out except at her periods.

### Drawing-room Meetings.

### THE ROYAL FREE HOSPITAL.

THE formation of a Ladies' Association occupied the attention of a large number of ladies (among whom were a few men) on Thursday afternoon, May 30th, at 13 Carlton House Terrace, the residence of Sir Edwin and Lady Durning-Lawrence, at whose invitation the guests assembled to meet Princess Christian and her daughter, Princess Louise of Schleswig-Holstein.

The meeting was not a long one. Mr. Charles Burt, chairman of the Royal Free Hospital, introduced the subject, pointing out especially why ladies should assist, and why the hospital was in need of help at all. Nearly three millions of poor persons, he said, had benefited in one way or another since 1828, when the hospital was founded. For a long time the directors had felt that, situated as it was, away from the West End, a good deal had been lost that other hospitals had secured, and it was desired to amend that state of things by means of the proposed Association. The rules, a copy of which was in everyone's hands, had been based upon those of a similar society; on the committee of one such society, he said, was an energetic lady, without any influence, who had obtained £25,000. Annual subscriptions, he continued, were greatly needed; the expenditure exceeded the income by £1,000; and it was simply impossible to carry on the work without more help. Nothing was more powerful than a woman's influence, and the work in which he invited them to share was a Christ-like work. He hoped that this was the beginning of a new era for the Royal Free Hospital. He proposed that the Association be formed.

Mrs. Garrett Anderson seconded the proposal. She said she thought that there were many young women ready and anxious to do something for the hospitals, but they did not quite know how to begin. This association would show them the way. She was heartily glad that such an organisation should be formed to help the hospital whose doors were thrown open to medical women when those of others were closed against them. Lord Stamford proposed, and Miss Wedgwood seconded, that the Princess Louise be appointed president; the latter said that the Princess had already brightened the wards with her presence. Other speakers were Mr. Layland Barratt, Mr. Henry Silver, the Vicar of Clerkenwell, Lady Bruce, and Lady Llangattock. At the close of the meeting Princess Christian said she was much obliged for the hearty welcome given to herself and her daughter.

The following ladies were elected vice-presidents:—The Countess of Stamford, the Countess of Cavan, the Lady Reay, Lady Llangattock, Lady Durning-Lawrence, Lady Owen Roberts, the Hon. Mrs. Whitbread, the Hon. Mrs. Tallents, Mrs. Garrett Anderson, M.D., Mrs. Scharlieb, M.D., and Mrs. Jessel.

### NORTH-EASTERN HOSPITAL FOR CHILDREN.

BY permission of Emily, Lady Ampthill, a drawing-room meeting was held, on Tuesday afternoon, at 19 Stratford Place, in aid of the building fund of the North-Eastern Hospital for Children. The proceedings were considerably enlivened by a couple of songs charmingly rendered by Miss Martha Cunningham. The long, shady drawing-room was quite filled with people when the Treasurer of the hospital took the chair. It was not his intention, he said, to detain the meeting by a long speech. He only wished to impress upon them, with all the emphasis in his power, the dire need of the hospital for funds, failing the receipt of which they would speedily find themselves involved in great difficulties.

The Duchess of Somerset, who was the next speaker, said that they wanted to more than double the subscriptions and place the hospital on an entirely new basis. The sum they were asking for was £20,000, and this seemed a very large

### DRAWING-ROOM MEETINGS.—*Continued.*

amount, but if they would help in every way that lay in their power it would soon be forthcoming. In these busy days it was difficult to find time to do all that we wished, but at any rate everyone could make an effort to enlist the sympathy of others and help the West End to realise the absolute necessity for a much larger hospital in the North-Eastern district. At the present time for lack of adequate room the hospital was compelled to refuse many urgent cases; £11,000 was needed to finish the first block of buildings, but the total sum asked for was £20,000, and this they earnestly hoped they would get before the Coronation season was over. It would, indeed, be a most fitting act of thanksgiving for the mercies of peace to do something towards so laudable an object. Many people who were unable to help with gifts of money might yet assist largely by their personal influence and service.

Mrs Josceline Bagot informed the meeting that she had done all that lay in her power to become acquainted with the needs of the hospital, and considered that it was one of the most deserving cases she had ever seen. The enlargement of the hospital was an imperative duty to the children of the north-east of London. It was only necessary to see the neighbourhood and the thousands of puny ill-fed children to realise how utterly inadequate 57 beds were. Owing to the continued call for more beds, children were constantly obliged to leave the hospital before they were fit to do so. There was an equal demand on the resources of the out-patients' department, there being from 300 to 400 surgical and 80 casual cases a week. As to the management, Mrs. Bagot said that she was immensely struck with the efficiency of the whole hospital, which had even got the light treatment for cases of lupus. The matron had given an unconscious tribute to the nurses and also to herself when she said that she had been at the hospital for 25 years and in that time had only known two or three nurses who had not been satisfactory. Additional rooms for the nurses were urgently needed.

At the conclusion of the proceedings the Chairman stated that the Duchess of Somerset had handed him a £10 note. Other gifts were also received before the meeting dispersed

### Workhouse Infirmary Nursing Reform.

BY SOLOMON C. SMITH, M.D.

(*Continued from page* 123.)

### The Question of Supply.

A great deal is made by many, and especially by some who are most intimately concerned with this question of workhouse nursing, about the difficulty of finding the nurses even if the conditions were made attractive. We are told that the nurses do not exist, that it is not a matter of pay or treatment or popularity, but that really the nurses are not to be had, and that some special means must straightway be adopted for increasing the supply. And if one could believe that all difficulties could be swept away to-morrow, and that one would have to find the nurses the day after, I would agree. But reform is a slow and gradual process, and by the time the workhouse service is made attractive to nurses I feel sure that the nurses will be ready.

### Suggested Reforms.

What then should be done to make the nursing service sufficiently attractive and so to secure efficient attendance upon the sick in workhouses, without at the same time entailing prohibitive expense upon the ratepayers? First of all, I think we shall be right in laying it down as a broad principle that, wherever there are sick there should be properly skilled nurses. It cannot be right that while one pauper is supplied with the best of nursing, another, merely because he happens to be in a small union, should be without skilled nursing altogether. Surely it is in the small workhouse, far away from medical help, that skilled and experienced nursing is wanted more than anywhere; yet that is just where it is often entirely absent. Whether a workhouse is big or little, if sick people are to be dealt with I think there should be at least one trained nurse on the spot.

### Three Groups.

Starting then on this principle we see at once, if we look into the condition of affairs as settled by the Order of 1897, that workhouse infirmaries may be divided into three distinct groups: (1) the separate infirmaries with resident medical officers, with which just now we have nothing to do; (2) medium-sized workhouses in which there are superintendent nurses as well as the ordinary nurses, who, according to the Order, must be "experienced" but may be untrained women; (3) small workhouses in which there are no superintendent nurses, *i.e*, in many instances, no trained nurses.

### Medium-Sized Workhouses.

In regard to (2) the matter seems to me to be comparatively simple. We have but to apply the principle which is found to be in practical operation in regard to almost all forms of labour, namely, that if work is to be done well it must be done under supervision. Whether we build a house, or spin cotton, or make a lady's dress, while the actual work is done by one set of people, it is supervised by another. The foreman or the forewoman is the key to all industrial work, and this the Local Government Board recognised when they provided that whenever more than a certain number of paid servants were employed in attending to the sick in workhouses there should be a "superintendent nurse," who should be a thoroughly trained one—in other words, a nursing "forewoman." Unfortunately, the Order has not been accepted in this sense. This has been partly the fault of the guardians, who wanted to have their money's worth, but perhaps the nurses may also have been a little in fault themselves. It has become almost an axiom among trained nurses that an untrained woman can do nothing, and thus the tendency has been for the superintendent nurses to do the nursing instead of seeing that it was done. But if one considers what actually goes on in the wards, not of a London infirmary which, owing to the growing practice of removing from their homes almost all serious cases of acute disease, is practically a hospital, but in a country workhouse where most of the cases are chronic, aged or infirm, one sees that, although it is always necessary that skill and experience should be at hand, a very great deal of the work is what can be done well enough by untrained women (that is untrained in the modern sense), if only their work is constantly superintended by someone who knows what is what. One thus sees that the work of superintendent nurse should be that of really superintending the work of others rather than doing the work herself, and one thus comes to the conclusion that the post of superintendent nurse, if once freed from the incubus of the matron's interference, and if done under direct responsibility to the attending medical officer, would really become work of a superior and very responsible kind, and far more suited to ladies of some education, and it may be added of some age, than to the younger sort of nurses fresh from the training school whose professional fervour is, I fear, at the root of a

### WORKHOUSE INFIRMARY NURSING REFORM.—*Continued.*

good many of the difficulties which have arisen. My solution, then, of the problem, so far as regards the medium-sized infirmaries, is that the position and the independence, and the direct responsibility to the medical officer, of the superintendent nurse should be so far ensured as to attract to the service middle-aged nurses, and especially ladies of education. Such a post, with a fixed position, with her own rooms, with practical independence, with no menial work to do, and with all her time spent in supervising, in teaching, and in doing good, ought to be very attractive to many thoroughly trained nurses approaching 40 years of age, and especially to lady nurses who, after a dozen years of knocking about, are anxious for a home. The salary might be small, but as the advantages became known I feel sure that the number of candidates would be very large.

### THE SMALL WORKHOUSE.

The most difficult problem of all is what to do with (3) the small workhouse in whose sick wards there may perhaps be only three or four sick.

This is a serious matter. There are scores of small workhouses in which the number of sick does not exceed half a dozen, and the guardians hold that it would be throwing the ratepayers' money away to engage a fully-trained nurse to attend to these people, perhaps not one of whom, even for weeks together, may be seriously ill. And the Guardians are not altogether wrong. Moreover, all experience teaches us that a newly trained nurse deteriorates very rapidly when placed in charge of a small number of cases among which even the utmost enthusiasm cannot discover anything really "interesting." Yet at any moment some acute and serious complication may arise, and it cannot be right that even this half dozen should be left without any skilled care in their extremity. Now here we come to what I think is the key to the difficulty and its solution. What we want to do is to find useful and interesting and constant employment for our superintendent nurse even in the smallest workhouse. I say, Hand over the aged and infirm to her care. This would be a step in the direction in which I believe some members of the Local Government Board have long wished to move, namely, in the direction of the better classification of the indigent poor in workhouses. The Guardians have two very distinct duties, first to deter the able-bodied poor from becoming paupers, second to help and succour the sick, the aged, and the infirm. Not only the whole machinery, but the whole habit of mind which is found most useful in carrying out deterrent duties, is diametrically opposed to that which is found most effectual in performing the charitable and merciful *rôle* of succouring the helpless and the sick. Let us then divide them; let us in each workhouse keep the master as master of the house with full authority from one Board meeting to another (this is essential for the maintenance of order), let the matron look after the servants and the female inmates, acting, so far as they are concerned, as she now does, as the deputy of the master, and let these two have the absolute control of the deterrent half of the workhouse.

### THE POSITION OF THE SUPERINTENDENT NURSE.

But in regard to the sick, the aged, the infirm, and such of the imbeciles as shall be transferred to her care by the medical officer, let the superintendent nurse have charge; let her be the deputy of the master in all that concerns house discipline in her department, and the deputy of the doctor in all that concerns the carrying out of treatment; and let both master and superintendent nurse report to and take orders from the Board. Then, I think, things will go on well; a new class of superintendent nurses will come into existence, older, more experienced, perhaps a little less inclined to take offence, perhaps a little less likely to excite jealousy; and even in the smallest workhouse the superintendent nurse, having charge of a not inconsiderable number of persons, will have plenty to do, while in the larger ones she will become a very important official.

### THE AGED AND INFIRM.

If one considers the very extreme differences which exist at the present time between the character of the inmates of the sick wards in different workhouses, one cannot but feel that a great injustice is being done to a large number of the aged and infirm. In some cases we find that the sick wards are largely filled with these infirm and aged people, while in others we find that not only are the aged and infirm still kept in the workhouse proper (that is under the deterrent *régime*), but that a not inconsiderable proportion of the sick also are so dealt with. This must be wrong. The fact is that it is very difficult to draw the line between the infirm and the sick, and the best solution of the difficulty is to place them all on the charitable side of the house, thus at the same time removing a good deal of the excuse which now exists for dealing tenderly, perhaps more tenderly than he deserves, with the able-bodied pauper. If this wider division between the deterrent and the charitable work of the Poor Law should be found to work well, I feel sure that an enormous benefit would be conferred upon a large number of aged and feeble and thoroughly deserving people who at present are debarred from experiencing the gentler aspects of Poor Law relief by the fact that they have not yet had the good fortune to fall sick.

### A Nurse's Life in India.

BY ONE WHO HAS TRIED IT.

A NURSE'S life in India is not what many people might imagine it to be. Those who go out either to appointments or on speculation, unless they go with the resolve to fight through many things that are both hard and unpleasant, had better give up the idea. A nurse who leaves England for India also leaves a great deal of her life behind her. It is a lonely position. She has to be cut off from her relations, friends, and all the interests in which she has lived. She has to take all her courage and strength into both hands, and go forward to a land quite strange to her, with the hope, wish, and intent to do her best in the work she goes to undertake.

### THE VOYAGE AND CLIMATE.

First, there is the voyage. She is thrown into immediate contact with utter strangers, some of whom are kind and friendly, probably feeling that "one touch of nature makes the whole world kin"; others may be the reverse of friendly, though that is rare. Some are returning to the East, and are ready to tell about the country and its ways of living, and often to depict horrors much worse in anticipation than in reality. On arriving in India one feels bewildered, as the life is absolutely different in all respects. The changes in climate are most severe and trying—great heat, heavy rains, and a few months of very cold weather, as they have in Bengal or Punjaub. These latter months are welcomed by all, except the natives, who are very wretched, not having the intelligence or capacity for making themselves comfortable. The native servants are, as a rule, troublesome, and as at first the new comer has no command of the language, it is difficult to make known her wants. Gradually

### A NURSE'S LIFE IN INDIA.—*Continued.*

it becomes easier, and the feeling of newness and strangeness wears off.

### HOSPITAL NURSING.

Of course in hospitals under Government a home is practically provided for the sisters appointed to the service, with many allowances and regular monthly pay. The position socially is good, and as long as the workers keep their health they can live very happy, useful lives. If, on the other hand, a sister gives way to depression, and allows the hot weather to affect her, when houses are shut up all day, and when all exertion and work get to feel irksome, then things will be different. There is nothing like work to make one forget oneself, not setting aside the fact that it is physically impossible to work as hard and as fast in the hospital wards as one does in England.

### PRIVATE NURSING.

There are private nursing associations in India where a staff of nurses are kept, but as a rule they are not quite in a flourishing condition through lack of funds. The nurses are mostly trained in India. Generally speaking, they are Eurasians, and their number is limited. The supply is not equal to the demand, and I cannot help thinking that there is a large opening and wide field for private nurses who might work on their own account. But there are many things required to make that nurse suitable. To begin with, she must be a woman of education, well-mannered, earnest, energetic, and tactful, with letters of introduction to people of good social standing; also be connected with them both in India and England. She must be a woman with heart, brain, strength, and courage to go through a good deal of disheartening work at the beginning. When once she has established a good footing between herself and some of the foremost medical men she would assuredly win success. She would, of course, have to start with some money in hand. She might get sufficient to start a nursing home, keep it going, and be ever ready to supply a really good nurse when required. It would take a year or two to accomplish this, but the right woman in the right place could and would do it I feel certain. Funds are the first requisite: if they can be supplied to start and keep the home for some months it would soon pay.

### THE PATIENTS.

The work in hospitals goes on in much the same routine as at home. Hours of duty vary, but usually in the hot weather the day for a nurse begins soon after sunrise. There is much work to be arranged, as of course each day brings its own duties. Diseases are similar to those at home, such as heart cases, phthisis, pneumonia, rheumatism, etc., but those predominating in India are enteric and dysentery. The temperature of an enteric patient runs to a great height very often, and distress is greater owing to the heat and climate generally. Dysentery cases suffer much, and are frequently obstinate to treat, because the patient usually only goes to hospital when he feels very ill—not at the beginning, as he ought to do. Liver abscess cases are numerous.

### SPECIAL DIFFICULTIES.

A nurse's ingenuities are taxed to preserve ice and use it judiciously and without waste. Milk has a tendency to go wrong even though boiled, so care must be taken that cans and vessels are scrupulously clean. Servants have their work portioned out to them: they dust, sweep, clean, wash up, and cook; but all their work needs close inspection, as they have little or no idea of doing anything thoroughly. Punkahs in Bengal are worked from about April to October; but, of course, in the very hot months, unless doors are shut early—about 9 o'clock—the punkah only adds to the heat and distress of the sick.

### ALLEVIATIONS.

For a month at the beginning and end of the punkah season doors can be opened with greater comfort. The temperature of wards sometimes rises to 103°, though every means are used to keep them cool. But as a rule where wind is favourable for the tatties they can be kept down to 80°, which is pleasant. Kus-kus tatties are used in the doorways, are movable, and placed on the side of the house or ward from which the wind blows. They are screens made to fit the doorway, of coarse fibre called kus-kus. It has a peculiar pungent odour, and when thoroughly wetted by coolies kept for the purpose the hot air passing through the wet fibre causes a delightful coolness and decreases the temperature of the ward considerably. One can readily see that if if not kept wet they are useless, and as the tattie coolie is not a conscientious man, if he can curl himself up in a corner and sleep he prefers to do so. The day duty of a nurse in hospital ends about 8.30 P.M., with, of course, stated hours for meals and recreation. A night nurse begins work at 8.30 and is on duty till relieved at 6 or 7 A.M., according to the season or time of year.

### THE SOCIAL SIDE.

The English community in India being comparatively small, people are kind, especially so to nurses, and invitations are given to all entertainments in the station, so that when she is not working a nurse can enjoy herself thoroughly and make many friends. The management of the house and servants devolves on the matron or head sister, who has not only to keep a correct account of everything with a view to each sister taking her share of expenses, but she has to know and arrange hours of duty for each sister, visit wards, and if necessary, take her share of nursing work when short handed. Of course this latter arrangement prevails where the nurses live in quarters and have allowances made to them for their keep, as is usual in Government employment.

### Where to Go.

SATURDAY AFTERNOON, JUNE 21.—Midsummer Festival in aid of the Building Fund, at the Hospital and Home for Incurable Children, Maida Vale, W.

### To Nurses.

**WE invite contributions from any of our readers, and shall be glad to pay for "Notes on News from the Nursing World," or for articles describing nursing experiences, or dealing with any nursing question from an original point of view. The minimum payment for contributions is 5s., but we welcome interesting contributions of a column, or a page, in length. It may be added that notices of appointments, entertainments, presentations, and deaths are not paid for, but that we are always glad to receive them. All rejected manuscripts are returned in due course, and all payments for manuscripts used are made as early as possible after the beginning of each quarter.**

### TRAVEL NOTES AND QUERIES.

KNOKKE (Truth).—The office of the General Steam Navigation Company is 33 Great Tower Street. The boats sail from Tower Bridge three times a week, Tuesday, Thursday, and Saturday, but you should write for particulars, as hours and days change to suit tides. Knokke is already more expensive than it was, but it is still cheap. Write to Hôtel de Bruges for terms; if full try Hôtel de la Marine.

### Annual Conference of the Matrons' Council.

### PAPERS AND DISCUSSIONS.

THE fourth annual conference of the Matrons' Council of Great Britain and Ireland was held in London on May 29th and 30th. Papers on nursing topics were read, and discussions held on the first day, whilst the following day was chiefly given up to the consideration of State registration for nurses.

Miss Isla Stewart, matron of St. Bartholomew's Hospital and chairman of the council, opened the proceedings on May 29th. She alluded to the loss sustained by the council in the death of Miss Shirley, and briefly reviewed the work of the society during the past year, especially with regard to the sending of a delegate to the Buffalo Congress, communications with the Local Government Board concerning Poor Law infirmary nursing, and the formation of a society, under the auspices of the council, for the promotion of State registration for nurses.

Dr. Macleod Yearsley then read a short paper on the "Treatment by Heat of Chronic Ear Disease." He explained that this comparatively new treatment is of service in certain cases of deafness caused by chronic tympanic catarrh and middle ear sclerosis; for, as in some affections of the larger joints, hot air is capable, when properly applied, of breaking down adhesions in the middle ear and setting free again the chain of auditory ossicles to do their work. He exhibited the apparatus used—a copper chimney and funnel heated by a gas flame and provided with a thermometer, and an asbestos sleeve, which fits round the patient's ear. The air is used at a temperature of 400° Fahr., and the treatment continued for about 20 minutes at a time. This is followed by massage of the middle ear by means of a pneumatic arrangement, which was also exhibited.

Miss Todd, matron of the National Sanatorium for Consumption, Bournemouth, read a paper on the "Nursing of Tuberculosis." She considered the proper proportion of nurses to patients in sanatoria as 1 to 10, and detailed the staff for an institution of 100 beds, suggesting schemes whereby time spent in a sanatorium might be included in a nurse's training or regarded as preparatory to it.

The best methods of cleaning, furnishing, ventilating, and lighting sanatoria were described, and the nurse's work considered in detail, stress being laid upon the influence of her personality in persuading patients to take food. The taking of temperatures per rectum was shown to be difficult in institutions where the separate principle is not in force. The disposal of sputa was considered; samples of pocket and bedside spittoons were exhibited, a cardboard pocket contrivance like a cigar case being condemned. Paper handkerchiefs were shown, and also a linen bag with separate compartments for flask and handkerchief.

District workers had their attention called to some pamphlets published by the National Sanatorium for circulation amongst consumptives. A warning was given as to the disposal amongst the poor of the clothing of phthisical persons unless previously disinfected.

Miss Mollett, matron of the Royal South Hants Hospital, then read a most interesting paper on "Nursing Ethics." She defined ethics as good manners, and showed that the attacks lately made upon nurses referred more to their manners than their professional ability. Just as any lapse in morality is especially noticed in a clergyman, so details of conduct which would pass unnoticed in a non-professional woman are severely blamed in a nurse.

A nurse's character is formed for good or evil before she enters her training school, and coming from all ranks of society she must be expected to possess the manners of her class.

A picture was drawn of a private patient to whom were sent two equally capable and conscientious nurses; the one was considered cold and unsympathetic by the patient's friends, because she attended strictly to work and refused to gossip, whilst the other roused their jealousy by winning the patient's affection.

Many of the complaints now ventilated have originated through the patient begging a nurse to keep some fussy relative out of his room. She does this without explaining the reason, but at the expense of her own popularity.

In a hospital, nurses are taught to exercise a certain moral discipline in their wards, but this is the last thing private patients or their friends understand and appreciate.

Miss Mollett's paper gave rise to a keen discussion. The advisability or otherwise of a distinctive dress out of doors for nurses was much canvassed. The general feeling of the matrons present was decidedly against it, Miss Stewart pointing out that it simply draws attention to the wearer, has ceased to be any protection, and is frequently to be seen on undesirable persons.

---

### THE SOCIETY FOR THE STATE REGISTRATION OF NURSES.

Miss Louisa Stevenson, who had promised to preside at the meeting on Friday, was prevented by illness from doing so, and Miss Stewart took her place. She described the formation of the society, now numbering 460 members, and read a letter from Miss Stevenson accepting the presidency, and strongly advocating the principles of the society.

Miss Stewart showed that the nursing community have no central authority, no standard of ethics, and no powers of intercommunication. During the last ten years many hospitals and nursing associations have organised societies of their nurses, but these have not yet combined together. At present any woman can call herself a nurse, and at least five out of every six women sent out as nurses from institutions in London are inefficient. The Matrons' Council had therefore originated this society to procure State registration through Parliament, and to teach the nursing community and general public the need of an examining body to determine efficiency, and a governing body to define nursing ethics and control the profession.

Miss Mollett referred to an article written by herself 14 years ago on the necessity of State registration, and said it was even more required to-day. She quoted Sir H. Ackland, who in 1860 urged legal recognition of nurses; and also Sir James Paget, who considered it would be beneficial. She thought it wrong that there was no means by which the public could tell if a nurse was properly trained, and hoped inefficient women would be squeezed out of the profession.

Mrs. Groves stated that the demand for trained nurses in the provinces is far greater than the supply, hence the frequency of untrained women. She gave examples of inefficient nurses and also of the misdemeanours of trained nurses, showing the necessity of a controlling body on the lines of the General Medical Council.

Miss Burr, from the League of St. John's House, pointed out that the society she represented had pledged itself to State registration. She felt the necessity for educating nurses as well as the public on this subject, and hoped that personal feelings and prejudices would not be allowed to hinder the work of the new society.

Mrs. Bedford Fenwick regretted that the R.B.N.A. did not at present support State registration. So far very few of the large Metropolitan hospitals had joined the movement, but she thought this was because the nurses themselves had not been approached on the subject.

Mrs. King Roberts considered the creation of some central authority, to whose jurisdiction incapable and undesirable nurses could be referred, essential to the welfare of the general public. Hearing that a large section of the nursing world is opposed to State registration, she had hoped to have heard their views, and she much regretted that they had no representative amongst the speakers.

After the usual votes of thanks, the proceedings terminated.

### Everybody's Opinion.

[Correspondence on all subjects is invited, but we cannot in any way be responsible for the opinions expressed by our correspondents. No communication can be entertained if the name and address of the correspondent are not given as a guarantee of good faith, but not necessarily for publication. All correspondents should write on one side of the paper only.]

### NATIVE INDIAN MIDWIVES.

"A Member of the Indian Nursing Service" writes: I have been very much interested in your article of last week on native Indian midwives, and I quite endorse all your Bombay correspondent says. Child marriage is said to be one of the greatest curses of India; but at the least on an equal footing, stands to my mind, the native midwife (dhi); and I feel sure that every nurse, who has had anything to do with gynæcological cases in this country will agree with me. Their practices are barbarous, and they seem to be totally devoid of any sense of wrong-doing. If called in to a first labour case, or to one inclined to be at all prolonged, the "dhi," if she is allowed her own way, will very likely run the patient up and down the room, till she falls from exhaustion. Gentle exercise has no place in their consideration; another favourite method is to suspend the patient to the roof (the rooms are fortunately not very lofty as a rule) by a stout piece of cloth passed under the armpits. Excessive hæmorrhage, following directly on the birth of the child, is supposed to be absolutely necessary to the well-being of the patient; consequently if this is not sufficient to satisfy the "dhi," the patient is stood up against a wall (held in position if necessary), while she retires to the furthest extremity of the room, takes as good a run as the length of the room will allow, and butts her head with all her strength into the patient's abdomen; this process is sometimes gone through two or three times. The result is usually everything she could desire. In some families the relations of the patients will happily not allow this treatment, but in up-country towns it is more often the rule than the exception. I am alluding only to self-constituted native midwifery nurses; so far as I can speak from personal experience of those women who have been brave enough to enter our hospitals and go through their midwifery course in order to help their countrywomen, no praise of mine would be sufficient. On several occasions, which have come under my notice, when asked by the patient or her relatives to have resource to some cruel native expedient, she has refused, and has succeeded in bringing the case into hospital for further treatment. However regardless of personal cleanliness a native woman may be at ordinary times, she is, if in her own home, whether willing or not, given a very hot bath as soon as possible after the birth of the child. The exhaustion which supervenes, as a rule, and sometimes ends fatally, by no means induces the "dhi" to let her next patient have the benefit of rest and quiet. These usages have held sway among them for ages, and that is sufficient in their eyes to mark them with the "hall mark" of infallibility.

### NURSES' UNIFORM AND SUPPLEMENTARY TRAINING.

"Nurse Marion" writes: The abuse of the nurses uniform has, perhaps, a great deal to do with the abuse of nurses and their profession. In London we see the nursemaid, in full nurses' uniform, calling aloud to tram conductors and others; again, we see advertising agents in full nurses' uniform, distributing samples of quackery from door to door. Nurses, who have been discharged from hospitals for various offences, wear the uniform, and even the most degraded of our sex appear in it. To the majority of nurses it would be a relief if a special uniform, protected by Act of Parliament, could be assigned to them, a penalty being imposed on persons not authorised to wear it. This, however, should only be worn by nurses in training, and those who out of hospitals can produce certificates for at least a two years' course of training. Nurses discharged from hospitals, for any cause whatever before completion of training, should at once resign the uniform. There are at present many excellent nurses, holding two years' certificates, who find it impossible to procure positions simply because hospital authorities have chosen to extend the training to three years. I will presume that the three-year nurses are more proficient. Why, then, do not the hospitals help nurses who have proved useful to them in the past? Is it not possible that nurses holding two-year certificates could be appointed to vacancies as they occur, as charge nurses or ward-sisters, and so gain the additional year lost through no fault of their own? There are post-graduate and polyclinic hospitals abroad where deficient nurses can take a course of supplementary training from 6 to 12 months, for which they are awarded a post-graduate diploma. Why cannot this be arranged in England, with a salary of about £24 for the year? Further, I think that every nurse engaged in private practice should take a post-graduate course every five or seven years till she reaches the age of 45, and so keep conversant with all the new modes of treatment. It seems to me that this would to a certain extent prevent the overcrowding of the profession.

### THE INSTANT DISMISSAL OF PROBATIONERS.

"Justice with Mercy" writes: I have finished a three years' course of training. On looking back I feel quite disheartened at the amount of time that I had to give to ward cleaning, etc., instead of bandaging, sick-room cookery, and the many little things so necessary to the comfort of an invalid, as well as the great injustice of some of the sisters towards the probationers. As far as the committee were concerned they seemed to do all they could for our comfort and happiness, which we appreciated. Our bedrooms and sitting-room were comfortable. Our staff compared with many hospitals, was large, and had it not been for the continual instant dismissal of probationers we need never have been over-worked. A beautiful garden with a croquet lawn was provided. If only the sisters had been considerate and just, the pros. might have been very happy during their training. On more than one occasion the matron was very kind to me, although I have had one or two narrow escapes of being dismissed. Once I received a good shaking. I did not get a whipping, although if I had been told to prepare for one I must have obeyed or been dismissed in disgrace. We had three day sisters and one night sister. The injustice of two of the sisters was painful, and had it not been for the other day sister speaking up strongly for any pro. who had worked under her, very few, if any of us, would have got through our training. I often wonder what the matron and committee know of the characters of the sisters except that they are certificated nurses. The injustices I mean are the unjust reports of behaviour, work, etc., to the matron, thereby bringing on our heads punishment, and often dismissal, the extra work given near off-duty time making it impossible to an already tired pro. to keep her temper, and numerous petty trials of this kind. If a pro. is dismissed before her time she can only get into another hospital by favour. Is this just? She may have served half her time, and given satisfaction, yet, for the sake of one sister, she may be dismissed. A probationer had been with us seven months, doing well and giving satisfaction to the matron. She was a thoroughly conscientious and refined girl. A convalescent patient, a man who got up at 7.30 in the morning, going to bed at 8 o'clock in the evening, asked her when he was in bed for a bed-pan! She, being surprised, immediately asked the night sister, whom she expected would tell the man he was able to leave his bed whenever he liked. The pro. had charge of 39 beds under the sister. The sister told the pro. to give it! The pro. said that rather than degrade herself and the man in such a way, she would leave. She did so the next morning in disgrace for "disobedience." Under our regulations the matron has the power to dismiss instantly a probationer for disobedience or neglect. If this power is not used with more discretion, ought the matron to have it? Have we much chance of keeping refined and considerate if this is our training? How can our probationers get justice in this matter? In this particular case justice came to the pro. in a very unforeseen manner. About six months afterwards the sister, being engaged, left to be married. Just a week before she was to have been married she took a cold and a severe illness followed. On being told that she had not long to live, she asked to see the matron, who was immediately sent for, and on her arrival sister asked simply that she would have Nurse B. back to finish her training. Sister being too weak to speak much, matron graciously acceded to her request, and within three weeks our probationer was back in her work again, to the joy of herself as well as her fellow nurses. During the night sister took a change for the better, and gradually she got stronger and in time married. Is this an isolated case, or have other pros. had a similar experience without such a happy ending? That I may never become a stumbling block to any striving probationer is my prayer, for I intend taking sisters' duties in the hospital where I trained. During the afternoon my probationers shall sit down for one hour and spend their time in padding splints, making bandages, swabs, etc., as I have been allowed to do in one ward only. I never heard that any of the committee minded our sitting down to do any work that we could. We were quite as busy, and did our evening work all the better for the rest our feet got. When I am matron, and my pros. try to be disobedient to any reasonable order, I think I shall try a new punishment. For the first offence they will be sent to bed, for the second a whipping!

### Appointments.

[No charge is made for announcements under this head, and we are always glad to receive, and publish, appointments. But it is essential that in all cases the school of training should be given.]

Cambridge Infectious Diseases Hospital.—Miss Gertrude Hewer has been appointed charge nurse. She was trained at Todmorden Union Infirmary, and has since been night charge nurse at the Leith Joint Hospital, Astley, and the Monsall Fever Hospital, Manchester.

Farmfield Home, Horley.—Miss M. E. Hayes has been appointed sister. Miss Hayes was trained at Worcester General Infirmary, and since then has been on the staff of private nurses of the Queen Victoria Nursing Institution, Wolverhampton.

Florence Nightingale Hospital, Bury.—Miss A. M. Baker has been appointed matron. She was trained at Monsall Fever Hospital, Manchester, and the General Infirmary, Leeds. She has since been charge nurse at Brook Hospital, London; sister of children's ward at Bolton Infirmary; sister of male medical and surgical wards at Cardiff Infirmary; and since July 1900 sister of the female surgical ward at the General Hospital, Birmingham.

Hospital for Soldiers and Children, Devonport.—Miss E. Nicholls has been appointed matron. She was trained for three years at Fir Vale Infirmary, Sheffield, where she was afterwards assistant nurse for five months. She has also been charge nurse at the Park Hospital, London; super-intendent nurse at the Union Infirmary, Devonport. She holds the L.O.S. certificate.

Lincoln Workhouse Infirmary.—Miss Jessie Hancock has been appointed superintendent nurse. She was trained at Stamford General Infirmary and Queen Charlotte's Lying-in Hospital, London, and has since been nurse at Keighley and Bingley Joint Hospital.

Nantwich Union Workhouse Hospital. — Miss Catherine Clarke has been appointed superintendent nurse. She was trained at Paisley General Hospital, and has since been nurse at Manchester Maternity Hospital, and super-intendent nurse at Lincoln Workhouse Infirmary.

Passmore Edwards Hospital, Wood Green, N.—Miss Suie Leach has been appointed matron. She was trained at the Royal Bucks Hospital, Aylesbury, and has since been staff nurse at the Jaffray Hospital, Birmingham, sister at the Grantham Hospital, and for the last two years charge nurse at the Hospital for Women, Derby.

Skin and Cancer Hospital, Liverpool. — Miss Amy Ryder has been appointed charge nurse. She was trained at the Royal Southern Hospital, Liverpool.

Wantage Cottage Hospital.—Miss Frances Edith Pike has been appointed matron. She was trained at the General Infirmary, Burton-on-Trent, and has since been a private nurse at the Bristol and Clifton Nurses' Institution and Nursing Home, and sister of the male wards, Eastville Union Hospital, Bristol.

### The Nurses' Bookshelf.

From Cradle to School: A Book for Mothers. By Mrs. Ada S. Ballin, Editor of "Baby: the Mother's Magazine," etc. (Westminster: Archibald Constable and Co., Limited. Price 3s. 6d.)

Of books for mothers on the care of their children there is no end; but Mrs. Ballin's may claim a place for itself on account of its practical nature. While most writers agree that children's dress should be warm and loose, very few give details as to how it is to be accomplished, and parents, with the best intentions in the world, are often driven by sheer ignorance to fall back on the garments which have only the merit of traditional custom to recommend them. The minutiæ of Mrs. Ballin's instructions on this point may seem trivial to an ordinary reader, but they will be very welcome to many an inexperienced young mother. The same is true of her remarks on feeding, and the list of meals suitable for young children which she gives will be found very useful. On the other hand, we think that the advice given for the many ills which infant flesh is heir to is not always judicious. The prescriptions may be good, but what we object to is the assumption that the ordinary mother, who does not even know how to feed and clothe her baby in health, will at once recognise the symptoms of every disease, and administer the right medicine. It may be necessary to give advice as to the treatment of convulsions and other diseases where a fatal termination may come before the doctor can be called in, but in the majority of ailments advice had better be confined to the question of nursing, leaving that of doctoring alone. It is right to say that the question of nursing is by no means neglected, but the very mention of drugs, even when the rider is added that they should not be administered except under medical supervision is a temptation to some women to experiment with these on their own responsibility. Mrs. Ballin's words on the choice of nurses for children are very wise and deserve attention. She protests against the employment of the uneducated and untrained nurse-girl for the important work of looking after a child, but points out that the hospital-trained nurse, whom wealthy people often choose instead, is not inevitably the right person for the task. It is quite certain that many women who would be admirable in sickness get very tired of the task of keeping and amusing a healthy baby. Besides a natural liking for children there must be patience, tact, and abundant physical strength, as well as a knowledge of the way to amuse and teach young children, which can as a rule be learned in a kindergarten. Mrs. Ballin strongly recommends the employment of properly trained lady nurses, but incidentally shows why they cannot be employed except by the wealthy, when she mentions cases where the nurse will not wheel a perambulator and objects to almost all the work connected with her charge. Mrs. Ballin's remarks on the child in his social relations are full of good sense, as are also her opinions on education, though perhaps these err in the direction of condemning only instead of pointing out the way to something better.

### Novelties for Nurses.

By Our Shopping Correspondent.

Messrs. Mühlens, of Bond Street, the head London emporium for the celebrated 4711 Eau de Cologne, intimate that it can now be obtained in small as well as large bottles, and thus be a most inexpensive purchase. The same firm produce also other delicious perfumes and accessories of the toilette, of quality as excellent as that of the 4711 Eau de Cologne.

### Echoes from the Outside World.

### The Conclusion of Peace.

On Sunday evening it was officially announced that terms of surrender had been signed at Pretoria, and on Monday morning the following gracious message from the King to his people was published:—"The King has received the welcome news of the cessation of hostilities in South Africa with infinite satisfaction, and trusts that peace may be speedily followed by the restoration of prosperity in his new dominions, and that the feelings necessarily engendered by war will give place to the earnest co-operation of all His Majesty's South African subjects in prosecuting the welfare of their common country." The terms were stated forth on Monday, in the House of Lords, by the Prime Minister, and in the House of Commons by the First Lord of the Treasury. The principal points of the agreement are—1. That the Boers lay down their arms, and recognise the King as their lawful Sovereign. 2. That Boer prisoners may return on taking the oath of allegiance. 3. That Dutch is to be taught, at the request of parents, in the public schools. 4. That civil government is to be followed by representative government at the earliest possible date. 5. That £3,000,000 are to be provided by the British Government to restock Boer farms, rebels and foreigners being excluded. 6. That all Cape rebels are to be disenfranchised, the leaders to be tried for high treason, but no death penalty to be exacted.

### Reception of the News.

Although the news was known to many on Sunday evening, the intimation posted outside the War Office being followed by the appearance in print at the Mansion House and the newspaper offices of the significant words, "Peace is proclaimed," it was not until Monday that the country formally, and the world at large, realised that the war which commenced on October 11th, 1899, was really over. Rejoicings on a small scale commenced directly, and in several churches in London and the provinces "God Save the King" was sung at the evening service on Sunday. But on Monday everybody manifested a desire to celebrate the occasion, and flags, bunting, chinese lamps, and fairy lights were in evidence alike in the most splendid and in the meanest thoroughfares. The public enthusiasm also displayed itself in the reception accorded to illustrious personages. The King, driving to the Levée through St. James's Park, had a tremendous ovation, and the Queen, driving in Hyde Park, was the subject of a similar demonstration. Cabinet Ministers attending a Council were also loudly cheered. The mention of the names of Lord Kitchener and Lord Milner always sufficed to provoke a salvo of applause. It is a matter of interest that the news of the Peace of Amiens was announced 100 years ago on Sunday evening; and that it was on Sunday evening, March 30th, 1856, that the intelligence of the close of the Crimean war reached London.

### The King's Birthday.

The trooping of the colour was held on Friday last for the first time since King Edward ascended the throne. Although his real natal day is on November 9th, His Majesty has elected to keep his birthday at the end of May the same time of the year that Queen Victoria's was celebrated. A very large concourse of people had assembled on the parade ground at the Horse Guards. They were rewarded by an impressive ceremony, and by the sight not only of the King and Queen, but of the Prince and Princess of Wales and two of their children; the Duke and Duchess of Fife, Princess Victoria and Princess Charles of Denmark, Princess Henry of Battenberg and her children, Princess Christian and her daughter, and the Duke and Duchess of Connaught and their daughters. Out of compliment to the regiment, to whom the King had promised to give their colours, His Majesty wore the uniform of the Colonel-in-Chief of the Irish Guards. The colours were blessed by the Chaplain-General of the Forces, and by the Rev. Cyril Forster, and the troops sang a hymn accompanied by the massed bands. After the presentation the troops marched passed in slow and quick time, His Majesty taking the salute. Throughout the whole of the proceedings the Queen sat with one of her grandchildren on her knee. Upon his return to Buckingham Palace the King presented medals to a number of the Grenadiers who had won them in the South African campaign.

### The Coronation.

The King received King Lewanika, the Central African monarch, at Buckingham Palace last week. How greatly Lewanika was gratified by the reception may be judged from the fact that subsequently he sent a cablegram to his son in Barotseland as follows:—"To-day is the day of my life, for with my own eyes I have seen and saluted my Chief, who, besides being a great King, is a kind man."

Among the distinguished Indian Princes who have arrived for the Coronation is the Maharaja of Jaipur, whose travelling arrangements excited the amazement of Europe. He journeyed from Bombay to Marseilles, and from Marseilles to Calais by special train, by special boat to Dover, and by special train again to London. He is accompanied by a suite of 125 persons. His baggage weighs 45,000 kgs., and he has brought with him provisions for six months, and an adequate supply of the sacred water of the Ganges for ablutionary and drinking purposes.

The Sultan of Perak, with a small retinue, arrived in London on Sunday morning at Liverpool Street Station, and was received, on behalf of the King, by Sir William B. Hamilton, Under Secretary at the Colonial Office. He wore a dark military undress uniform, resembling that of the Rifle Brigade, and a jauntily-poised forage cap. The chief minister of his native suite was dressed almost entirely in white.

### Philanthropic.

The interest in the Exhibition of the Home Arts and Industries Association increases year by year, and the exhibition which has just closed at the Albert Hall has been more than usually successful. It was visited by the Queen, accompanied by Princess Victoria and the Prince and Princess Charles of Denmark. The Princess Victoria was amongst the exhibitors, and received a gold star, the highest mark of recognition for general excellence which the society can give, for an embroidered cushion most beautifully worked. It was placed on the Sandringham stall, which was naturally a special object of attention, and some good wood-work was noticeable as well as some woollen goods woven from the wool of the Sandringham sheep. Amongst the exhibits of a novel character were a number of toys made by injured or invalid soldiers who had served in South Africa, and the Queen, upon learning the nature of these particular exhibits, bought several toys for her grandchildren. She also acquired at different stalls a number of frocks and little garments to give away again in charity. Another of Her Majesty's purchases was a bedspread embroidered on linen by seven little boys, inmates of the Maida Vale Hospital and Home for Incurable Children. The object of the Home Arts Union is especially to promote the well-being of those unfortunate people, deaf, blind, crippled, feeble-minded, or incurable, etc., who, by reason of their infirmity, are unable to work at any of the ordinary avocations of life.

### Recreation.

The contest for the Ladies' Golf Championship at Deal on Friday last resulted in a victory for Miss May Hezlet. It is not the first time that this lady has been successful, for in 1899 she won both the English and the Irish Ladies' Championships. The match was one of extreme interest. There was a tie at the eighteenth, and again at the nineteenth hole, so that it was not till the twentieth hole had been played that Miss Hezlet really had success within her grasp. Miss Neville, her opponent, also played in splendid style.

### A Book and its Story.

DR. MARGARET TODD'S NEW NOVEL.*

DR. MARGARET TODD'S new novel is distinctly clever, and its theme, the ever-recurring one of the woman who loves and the man who deserts her, is treated with a fine reserve, leaves a few things for the imagination and intelligence of the reader to define, which is unusual with many able writers of the day.

The story opens in sunny Provence. "The hotel stood boldly out on a terrace overlooking the Mediterranean. Away down below the waves broke on rugged rocks, and for miles on either side the sunny coast was fringed with fantastic weird-looking pines. It was a place little known to the ordinary English visitor, and in bygone times the building had been a monastery. . . . The great blue waves came rolling steadily in, but the mistral lifted the foam from their crests and chased it gaily eastward in a sunlit fountain of spray. A hedge of aloes stood out grey against the blue, and everywhere was sunshine, such sunshine as gladdens the heart and makes a man's pulses leap." Vera Carruthers, the heroine, is the young daughter and companion of her father, an elderly savant, absent-minded, and imperceptive to all, that does not aid the advance of scientific research. A second marriage had added neither to his happiness nor to Vera's well-being, and it was in consequence of the frequent interchange of hostilities between Vera and her stepmother that he had brought her away with him to St. Vincent when prosecuting some scientific inquiries which necessitated his presence at a marine station in the vicinity. Vera's stepmother had been chosen, apparently, by the law of contrasts: she was narrowminded and puritanical. Vera's own mother had been a Frenchwoman and an actress, and by faith a member of the Roman Church. Apparently her religious influence had not been brought to bear upon her child, who, to escape the intolerable and petty persecutions of her step-mother, had adopted the agnosticism of her father, with a little paganism of her own, thrown in. To him she was a capable and ever-willing assistant in his work. To madame, their hostess, she was *très gentille et très originelle*, but to her father she was more. She "has a gift for truth," he said, "that is rare in her sex. She says what she thinks and draws what she sees. She is drawing some of Brillat's slides and preparations for me. If ever she does research work of her own—as I quite hope she may—she will not be one of those of whom Darwin said, "Ah, I never read a page of him without thinking there's five or six years' work for anyone to see whether that's true." To Giles Willoughby, a young Edinburgh student who had arrived at St. Vincent in the wake of the Carruthers, sent there for a winter holiday to get through some "general reading" and to get rid of a cough which had taken hold of him, she appeared as "the quaintest figure of a girl" that he had ever seen, "with dusky hair that fluffed, it did not curl, in great clusters about her brow, her eyes, and her neck." And then "her wonderful eyes, with their mocking lights and infinite depths." Willoughby was fresh from the University, where he had carried off the blue ribbon and was pointed out by professors and students alike, as the most promising graduate of the year, and who had proved, by what to English ears sounds rather an incongruous combination, "that a medical student may teach in a Sunday School and go to prayer-meeting, and yet be able in case of need to punch a man's head." His home life had been that of the simple routine of a Scotch household. His womenkind were entirely unlike this fascinating, witch-like sample of the sex into whose company he had been so unexpectedly precipitated. Thrown into the presence of such magnetic attractions, feeling intensely the isolation which comes upon everyone more or less at first in a strange country, where no one speaks the language familiar from childhood and everything and everybody is new, he realised on the night of his arrival, for the first time in his short life, the desolation of it all, as he threw open the volets and looked out. "To the right a mystery of pine trees stood black against the sky. Giles shivered as he closed the window. . . . What a wind! already his little candle had guttered half away. Ignoring the comfortable armchair, he seated himself on his bed in a mood of inexplicable dreariness. For the first time in his life, with a force of realisation that was vivid as a lightning flash, he felt himself alone—absolutely cut adrift in space." The force of present surroundings is apt to make the best men oblivious of matters connected with those who are absent; and even the fact of an unposted letter to the Scotch girl at home who loved him, "the woman with the fair, untried face, like thousands more," whom he had chosen for his wife, failed to banish the demon of isolation for the time being. At this critical moment he is introduced to Vera by her father. "So Giles stood on the threshhold of the Enchanted Palace, enjoying its sunny exposure and believing himself proof against all its blandishments; for was he not armed with the triple steel of religion, loyalty, and common-sense. There are dangers to which a man of principle is not liable." Not many pages farther on we find Willoughby arguing with himself as to the possibility of breaking his engagement to the girl at home under the spell of Vera. "He thought of her first, however," and he could never settle in Edinburgh if he broke it off; and yet, to settle elsewhere would be professional suicide. His reputation was in a way made. Opportunities were awaiting him there that had only to be seized—and yet—yet—was there not Vera too? "What a flower on the tree of happiness she was! Only seventeen, a mere child, her heart was opening for the first time, and she felt as sure of him and his love—as other girls felt of God." Willoughby had not told her of his engagement. He came to her as a free man. And in the torture of remorse to which he was a prey he cried out, "Now is the time to turn back." Of course he tried to pray, but prayer seemed such a rusty, obsolete weapon in conflict with this new foe. "Oh God, I have striven to live uprightly and honestly, why has this come upon me?" "Did no voice from heaven say, "Because thou hast striven uprightly and honestly, therefore I call thee to a harder fight." If the voice spoke, Giles did not hear, he wanted Vera. So the days passed rapidly on, and then, after spending them in Vera's company, winning her confidence and her love, he tore himself away and left her to bear her fate as best she might. He returned to Edinburgh and to his *fiancée*, to find his father dying and the circle of the inevitable closing round him. His mother and sisters, too, left in his charge, and the arrangements for his marriage to be made. It all seems pitiable enough. He had written one letter after his return to Vera; a love-letter so impassioned that it satisfied even her—one of those rare letters that make a woman feel she could not have written it better herself. After that came tidings of his father's death, and then there had been but one letter more. The rest was silence. Her father had returned home and left her in charge of Madame, a kind motherly woman, and then, "in her dread loneliness, with no little kindly offices to offer him, with nothing to take her thoughts from her own sore position, she wonders if she is going mad. . . . "If I could only sleep just for one little night as I used to do." How Vera works out her redemption, how "the way of escape" from public knowledge of the error of her youth is borne, our readers will read for themselves, we hope, in this vivid study of a man of strong passions but weak will, and of the girl he deserted and betrayed, leaving to her the burden of a blighted life.

* "The Way of Escape." By Graham Travers. Margaret Todd, M.D. 1 vol. 6s. Publishers: Blackwood and Sons.

### For Reading to the Sick.

THE CHRISTIAN'S APPEAL.

SAVIOUR, and Suffering God! when I,
Knowing it is my time to die,
Upon my final cross shall lie;

When Nature's deepening shadows fall
O'er Soul and sense, and like a pall
Suit all things to the funeral;

In my eclipse, oh, let me see
Thy Sorrows, borne in love for me,
Upon Thy Cross, on Calvary—

Borne, that I might in dying rest,
And lay, undarkened, undepressed
My head on Thy all-loving Breast.

*C. J. Black.*

I have read that such plants as produce a cruciform flower are all alike free from poison.

Life has its blossoming season preparatory to its season of fruit.

And many lives by bereavement, disappointment, pain, hope deferred, blossom, so to say, with crosses.

A choice and blessed blossom, if it correspond with nature's emblem and harbour no venom. For one day the cross petals will drop off, and only the good fruit remain.

Christ, "for the joy that was set before Him, endured the cross, despising the shame, and is set down at the right hand of the Throne of God."

For our sakes the cruciform blossom of His mortal life was agony and shame; for our sakes the salutary fruit of His life immortal is glory and grace.

And now He looks down from heaven, from the habitation of His holiness and of His glory, if so be He may in us see of the travail of His Soul and be satisfied.

Once He looked, and there was no man. Once he looked, and one penitent went out and wept bitterly.

Now He looks on you, on me.

*Christina Rossetti.*

On the cross lifted
Thy face I scan
Bearing the cross for me
Son of man.

Oh, I will follow Thee
Star of my soul,
Through the deep shades of life
To the goal!

Yes, let Thy cross be borne
Each day by me;
Mind not how heavy if
But with Thee.

Lord, if Thou only wilt
Make me Thine own,
Give no companion save
Thee alone.

Grant through each day of life
To stand by Thee!
With Thee when morning breaks
Ever to be!

*E. Monro.*

### Notes and Queries.

**The Editor is always willing to answer in this column, without any fee, all reasonable questions, as soon as possible.**

**But the following rules must be carefully observed:—**

**1. Every communication must be accompanied by the name and address of the writer.**

**2. The question must always bear upon nursing, directly or indirectly.**

**If an answer is required by letter a fee of half-a-crown must be enclosed with the note containing the inquiry, and we cannot undertake to forward letters addressed to correspondents making inquiries. It is therefore requested that our readers will not enclose either a stamp or a stamped envelope.**

*Insurance.*

(75) Will you kindly tell me if there is any insurance other than the Royal National Pension Fund for Nurses wherein a nurse can insure against sickness, especially influenza?—*W. B.*

The Royal National Pension Fund for Nurses is the only association which insures against sickness.

*Post.*

(76) Will you kindly tell me to whom I should apply for a post on the staff of a Nursing Association in Japan or Italy?—*Sister.*

You should apply to the lady superintendent of the nursing association which you wish to join. The Doshisha Mission Hospital's District Nursing Corps, Kyoto, Japan; the Anglo-American Nursing Home, 265 Via Nomentana, Rome; and the Institute for Trained English Nurses, Via Roma, San Remo, are the three best known.

*Address.*

(77) I should be much obliged if you could give me the address of the Church Medical Mission.—*W. S. M.*

Do you mean the Medical Missionary Association? If so, the address is 49 Highbury Park, N.

*Analyst.*

(78) I should be much obliged if you would kindly give me the address of an analyst, and say if it is impossible for a woman to have her milk analysed, and so find out the cause why she cannot nurse a two months' old baby.—*E. M. A. S.*

Your medical man will see about that if you ask him.

*Disinfecting Bedding.*

(79) Would you please tell me if it is necessary to disinfect the mattress and the palliasse after a patient's recovery from typhoid fever?—*V. T. L.*

Most certainly. Send them to be disinfected by steam.

*Home for Infectious Cases.*

(80) Is there any medical home or private infectious hospital where a London district nurse or other lady worker could go for payment if small-pox, scarlet fever or diphtheria had been contracted?—*M. B.*

There are advertisements of private homes which receive infectious cases in the Nursing Section of THE HOSPITAL, but we doubt whether they would receive or be allowed to receive cases of small-pox.

*Etiquette.*

(81) Will you kindly tell me how a nurse corresponding professionally with a surgeon would address him at his private residence?—*Nurse Prudence.*

Just as she would at any other time, beginning "Dear Sir" and ending "Yours faithfully."

*The Red Cross.*

(82) In 1897 I entered the Mecklenburghischer Red Cross Society, and last year (1901) obtained the certificate, Mecklenburg Red Cross brooch, and badge for all German Red Cross nurses also. Unfortunately, mother wishes me to come to England and give up nursing in Prussia I find that all nurses here who leave, even when they are old and receive their pension, or leave to get married, etc., are obliged to give back brooch and badge to the committee. And I want to know if it is possible to obtain the Red Cross in England.—*M. A. B.*

Write to the Secretary of the Red Cross Society in England, at 5 York Buildings, Adelphi, London, W.C.

**Standard Books of Reference.**

**"The Nursing Profession: How and Where to Train." 2s. net; post free 2s. 4d.**

**"Burdett's Official Nursing Directory." 3s. net; post free, 3s. 4d.**

**"Burdett's Hospitals and Charities." 5s.**

**"The Nurses' Dictionary of Medical Terms." 2s.**

**"Burdett's Series of Nursing Text-Books." 1s. each.**

**"A Handbook for Nurses." (Illustrated). 5s.**

**"Nursing: Its Theory and Practice." New Edition. 3s. 6d.**

**"Helps in Sickness and to Health." Fifteenth Thousand. 5s.**

**"The Physiological Feeding of Infants." 1s.**

**"The Physiological Nursery Chart." 1s.; post free, 1s. 3d.**

**"Hospital Expenditure: The Commissariat." 2s. 6d.**

**All these are published by the SCIENTIFIC PRESS, Ltd., and may be obtained through any bookseller or direct from the publishers, 28 and 29 Southampton Street, London, W.C.**

